# Janus-faced Acrolein prevents allergy but accelerates tumor growth by promoting immunoregulatory Foxp3+ cells: Mouse model for passive respiratory exposure

**DOI:** 10.1038/srep45067

**Published:** 2017-03-23

**Authors:** Franziska Roth-Walter, Cornelia Bergmayr, Sarah Meitz, Stefan Buchleitner, Caroline Stremnitzer, Judit Fazekas, Anna Moskovskich, Mario A. Müller, Georg A. Roth, Krisztina Manzano-Szalai, Zdenek Dvorak, Alina Neunkirchner, Erika Jensen-Jarolim

**Affiliations:** 1 Comparative Medicine, The Interuniversity Messerli Research Institute of the University of Veterinary Medicine Vienna, Medical University Vienna and University Vienna, Vienna, Austria; 2 Institute of Pathophysiology and Allergy Research, Center of Pathophysiology, Infectiology and Immunology, Medical University of Vienna, Vienna, Austria; 3 Department of Anesthesiology, General Intensive Care and Pain Medicine, Medical University of Vienna, Austria; 4 Department of Cell Biology and Genetics, Palacky University, Olomouc, Czech Republic; 5 Christian Doppler Laboratory for Immunomodulation, Medical University of Vienna, Vienna, Austria

## Abstract

Acrolein, a highly reactive unsaturated aldehyde, is generated in large amounts during smoking and is best known for its genotoxic capacity. Here, we aimed to assess whether acrolein at concentrations relevant for smokers may also exert immunomodulatory effects that could be relevant in allergy or cancer. In a BALB/c allergy model repeated nasal exposure to acrolein abrogated allergen-specific antibody and cytokine formation, and led to a relative accumulation of regulatory T cells in the lungs. Only the acrolein-treated mice were protected from bronchial hyperreactivity as well as from anaphylactic reactions upon challenge with the specific allergen. Moreover, grafted D2F2 tumor cells grew faster and intratumoral Foxp3+ cell accumulation was observed in these mice compared to sham-treated controls. Results from reporter cell lines suggested that acrolein acts via the aryl-hydrocarbon receptor which could be inhibited by resveratrol and 3′-methoxy-4′-nitroflavone Acrolein*-* stimulation of human PBMCs increased Foxp3+ expression by T cells which could be antagonized by resveratrol. Our mouse and human data thus revealed that acrolein exerts systemic immunosuppression by promoting Foxp3+ regulatory cells. This provides a novel explanation why smokers have a lower allergy, but higher cancer risk.

Acrolein (2-propenal), the highly reactive, water-soluble α,β-unsaturated aldehyde is a strong toxic respiratory irritant. It is generated at all sites of incomplete combustion, like during domestic cooking with oil, wood burning, combustion of fuels and plastic, and in the body as a product of oxidative stress^1^. However, cigarette smoke is considered the major source of human exposure to acrolein^2^. Reports of the acrolein content in cigarette smoke vary depending on the type of cigarette and added glycerin making up up to 220 μg acrolein per cigarette^3^,^4^. As such the health impact arising from inhalation of acrolein is higher than those from other routes of exposure. An important aspect here is that cigarette filters have no significant effect on the composition of the side-stream smoke where acrolein usually resides, and which is inhaled by passive smoking^5^.

In this study we decided to especially concentrate on passive smoking. We established a mouse model mirroring passive exposure to acrolein as a major single compound, instead of using smoke extracts. The intranasal exposure route was selected due to the fact that particularly the anterior part of the nose seem to be the prime target for acrolein^6^. In dogs, who actually also are exposed by passive acrolein exposure, nasal retention of acrolein was about 80% of the applied dose. Therefore, only 20% of acrolein penetrated the nasal passages and reached the lower respiratory tract^7^. In passive smokers a higher percentage of it will thus be solubilized in the aqueous nasal secretions^7^, than in active smoking deeply inhaling acrolein via the mouth into the lower respiratory tract. The amount of acrolein solubilized at the nasal mucosa will therefore directly depend on the minute volume inhaled, time of exposure, but also on its environmental concentrations, which are in a (smoking) coffee shop 30–100 ppb; train 10–120 ppb; car with three smokers (windows open) 30 ppb (average); car with three smokers (windows closed) 300 ppb (average); and restaurant 3–13 ppb^8^. Acrolein rapidly enters tissue by passive diffusion and readily reacts with its electrophilic α-carbon primarily with SH-groups as well as primary and secondary amines^9^. The main metabolism route of acrolein occurs through formation of GSH adducts, leading to depletion of GSH. Acrolein mediated GSH adducts can also be catalyzed by glutathione-S-transferases. Further cleavage of γ-glutamic acid and glycine residues, followed by reduction results in its main metabolite 3-hydroxypropyl-mercapturic acid (HPMA), which is excreted primarily in the urine^10^. In humans, typical concentration of 3-HPMA in the urine are around 150 μg/L^9^,^11^ and 1200 μg/L^2^,^11^,^12^,^13^ in non-smokers and smokers, respectively. As such, Carmella *et al*. reported that smoking at least 10 cigarettes per day led to an average daily excretion of 1.7–2 mg or 7.7–9 μM HPMA. In comparison, one cigarette will generate roughly 60 μg or 1.1 μM acrolein^12^. Thus, in humans nearly all acrolein is absorbed primarily via the aqueous lining of the nose, is metabolized and excreted primarily via the urine in form of HPMA^10^.

In this study we aimed to specifically address the immunological impact of acrolein, at concentrations relevant in passive smoking. The rationale behind the study is the knowledge that tobacco smoking is an established risk factor for a large number of major diseases including cancer, but seemed to be protective against allergic sensitization, when allergy is not yet established^14^,^15^,^16^,^17^,^18^. The inverse correlation of smoking to allergic sensitization and cancer risk is striking and suggests that smoking may be causative in the aberrant immune regulation of both diseases. This is in line with AllergoOncology studies showing a negative correlation of allergy or atopy, and the risk for specific cancer types^19^.

## Results

### Inhibition of antibody-formation upon acrolein-exposure

**Figure 1:**
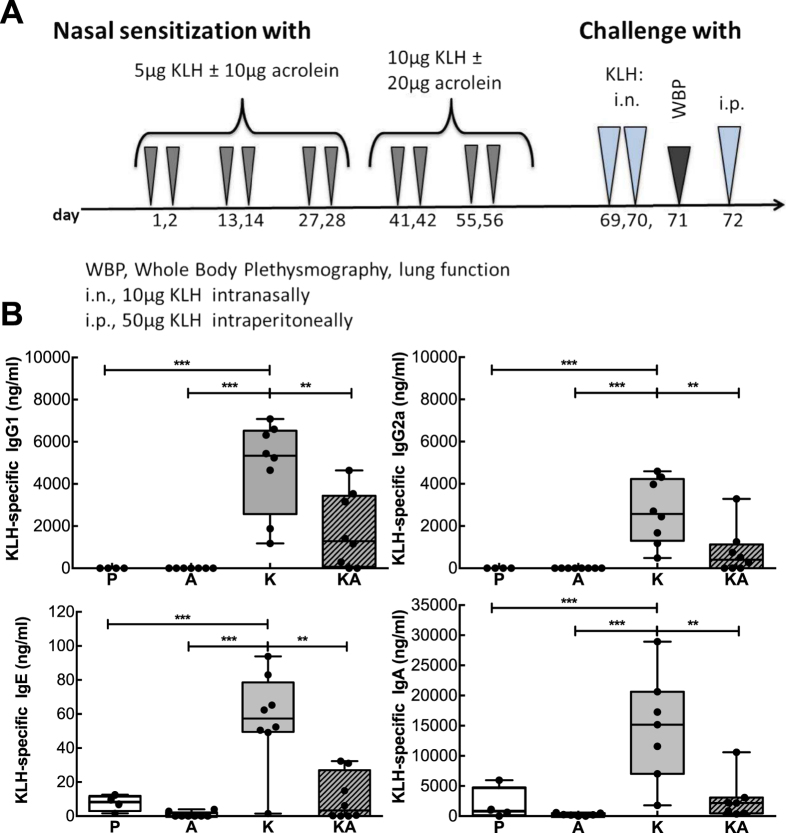
Inhibition of antibody-formation upon exposure to acrolein. (**A**) As depicted in the allergic sensitization scheme, mice were nasally sensitized 5 times in biweekly intervals with KLH and/or acrolein or PBS as control. After a nasal challenge, lung resistance was measured by whole body plethysmography (WBP). The following day KLH-specific anaphylactic symptoms, were determined by challenging mice intraperitoneally and recording rectal temperature. (**B**) KLH-specific antibody-levels in serum of mice nasally treated with PBS (P, n = 4), acrolein (A, n = 8), KLH (K, n = 8) and KLH in combination with acrolein (KA, n = 8). Representative data from two independently performed experiments are shown. Groups were compared by ANOVA following Newman-Keuls Multiple Comparison test. Mean ± SEM; **P < 0.01; ***P < 0.001.

Regular exposure to moderate amounts of acrolein was mimicked in a BALB/c mouse allergy model (Fig. 1A). Mice were sensitized via the nasal route to exclude any additional percutaneous sensitization such as during aerosol-exposure. It has been shown that upon passive respiratory exposure to acrolein up to 80% is solubilized at the nasal mucosa^7^,^20^. This was one of the major reasons for selecting the intranasal route in our mouse model. Further, we selected a single application of 10 to 20 μg acrolein per mouse corresponding to approximately 5 to 10% of acrolein generated per cigarette for the following reasons: The acrolein concentrations in air particles generated in relation to smoking were determined to be between 30–300 ppb^8^, and from each cigarette up to 200 μg acrolein is generated reaching the respiratory mucosa of an active smoker^3^,^4^. We estimated that in humans 30 min respiration at a minute ventilation of 7 l/min and at a particle concentration of 50 ppb, a dose of 20 μg acrolein would likely reach the nasal mucosa of passive smokers. Similarly in mice, considering a smaller mucosal area and minute ventilation of 29 ml/min, a dose of 20 μg acrolein would correspond to 60 min exposure to 5 ppm, which is a common concentration used in animal studies^21^,^22^,^23^ that - in this time interval - does not lead to pathophysiological changes^24^.

For our proof-of-concept studies we chose monomeric keyhole limpet hemocyanin, KLH, as immunogen as it easily evokes immune responses^25^.

Mice sensitized to KLH (group K) formed antigen-specific IgG1, IgG2a, IgE and IgA antibodies, but co-exposure of mice to acrolein (group KA) led to a highly significant reduction in the production of all these immunoglobulins classes to the otherwise very immunogenic protein KLH (Fig. 1). In fact, levels of antibodies in mice sensitized with KLH in combination with acrolein did statistically not differ from the control groups treated with PBS (group P) and acrolein alone (group A). Taken together, acrolein greatly inhibited the generation of antigen-specific antibodies in our model.

### Impaired cytokine-responses in splenocytes of acrolein-exposed mice

**Figure 2:**
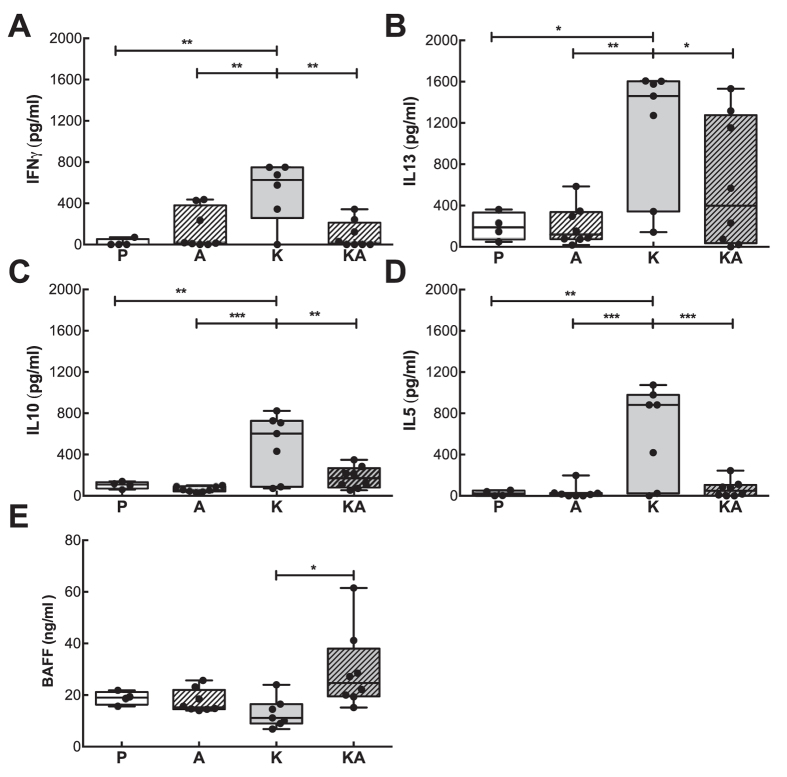
Reduced cytokine-response to KLH in mice exposed to acrolein during sensitization. Supernatants of splenocytes collected after 72 h stimulation with KLH (25 μg/ml) from mice nasally treated with PBS (P, n = 4), acrolein (A, n = 8), KLH (K, n = 8) and KLH in combination with acrolein (KA, n = 8) were stimulated with KLH for 72 h and analyzed for (**A**) IFNγ, (**B**) IL13, (**C**) IL10, (**D**) IL5 and (**E**) BAFF. Representative data from two independently performed experiments are shown. Groups were compared by ANOVA following Newman-Keuls Multiple Comparison test. Mean ± SEM; *P < 0.05; **P < 0.01; ***P < 0.001.

Next we addressed the cellular response of the different treated groups. Similarly to the declined KLH-specific antibodies of mice sensitized to KLH in conjunction with acrolein, their splenocytes showed a marked decrease in the secretion of IFNγ, IL5, IL13 and IL10 cytokines when compared to splenocytes from KLH-sensitized mice after stimulation with KLH (25 μg/ml, Fig. 2). Though KLH-sensitized mice displayed the most prominent Th2-response and released significantly higher levels of IL13, also splenocytes of mice sensitized to KLH in conjunction with acrolein secreted IL13, but at significantly lower levels than the KLH-only group. Hence the cytokine-formation was not entirely blocked by acrolein treatments. Overall, we observed a distinct decrease in the cytokine-response in mice sensitized to KLH in combination with acrolein.

Supernatants were also analyzed for B-cell activating factor (BAFF) as antibody-production was greatly diminished in the group co-sensitized with KLH and acrolein. In contrast to our expectations, significantly higher levels of BAFF were present in the supernatants of splenocytes from the group treated with KLH and acrolein (KA-group). The data imply that myeloid cells were able to respond adequately by releasing BAFF, but that acrolein affected in particular B- and T-cells.

### Decreased hyperreactivity upon co-exposure to acrolein

**Figure 3:**
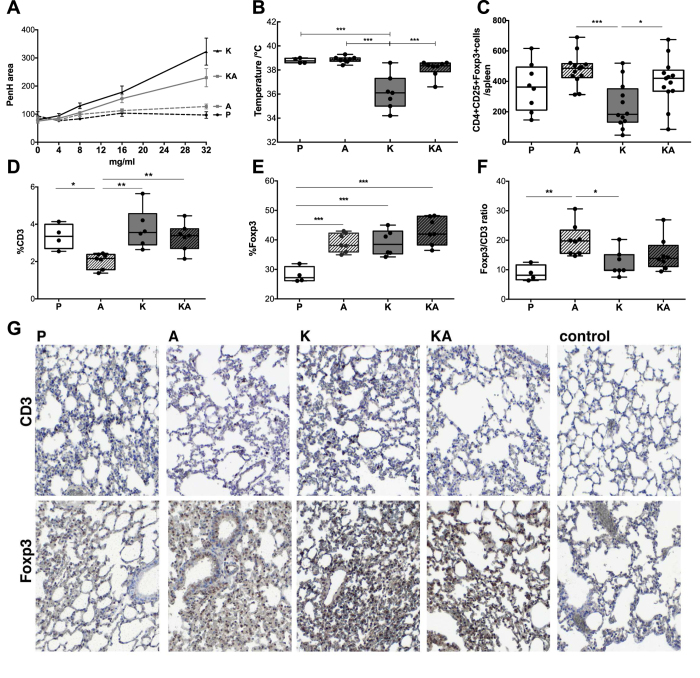
Reduced allergic symptoms in mice exposed to acrolein are associated with an increase in the Foxp3/CD3 ratio in the lung. (**A**) Mice nasally treated with KLH (K), acrolein (**A**), PBS (P) or with KLH in combination with acrolein (KA) were exposed to increasing doses of methacholine and the degree of airway hyperreactivity was measured by whole body plethysmography according to change in area of enhanced pause (Penh). (**B**) Rectal temperature drop 20 min after ip-challenge with KLH as a parameter to determine systemic anaphylactic reactions (**C**) Summary of CD4+CD25+Foxp3+ cell numbers counted in the spleens of the different treated mice of two independently performed experiments. (**D)** Whole lung sections were imaged by TissueFAX and positive stainings were quantified by Histoquest. Summary of immune-histochemical quantification % of CD3+ cells, (**D**) % of Foxp3+ cells (**E**) Foxp3/CD3 ratio of lung sections in the different treated groups. (**F**) Representative regions of lung sections stained for CD3 and Foxp3. 2 stained sections per animal were analysed by TissueFAX. Groups were compared by ANOVA following Newman-Keuls Multiple Comparison test. Mean ± SEM; *P < 0.05; **P < 0.01; ***P < 0.001.

We tested also the clinical reactivity of the mice in our allergy model after allergen challenge. In accordance with the marked decrease in the antibody as well as cytokine response, nasal challenge with KLH induced a significant increase in the lung resistance in the KLH-sensitized mice (K-group) only. The degree of airway hyperreactivity to increasing doses of methacholine was measured and expressed as change in area of enhanced pause (Penh). Co-sensitization with KLH plus acrolein in the KA-group resulted in a significant reduction in airway-hyperreactivity, almost reaching values of the groups sham-treated with PBS (P-group) or acrolein alone (A-group) (Fig. 3A). Similarly, systemic challenges with KLH led to a significant drop in body temperature only in the KLH-sensitized group, whereas addition of acrolein during KLH-sensitization protected mice from anaphylactic symptoms (Fig. 3B). The data collectively emphasize that acrolein impeded allergic sensitization by inhibiting antibody- and cytokine formation, resulting in reduced clinical hyperreactivity of the acrolein-treated mice.

### Foxp3/CD3 ratio in lungs of acrolein-groups is higher

Next we analyzed the immune cell composition in the spleens and lungs of the differently treated mouse groups. Significantly more regulatory CD4+CD25+Foxp3+ T cells accumulated in spleens of mice exposed to acrolein alone or in combination with KLH, compared to KLH-sensitized mice only (Fig. 3C). When we histologically quantified CD3- and Foxp3-positive cells of lung sections (Fig. 3D–G), an increased Foxp3 expression was observed in all groups (group K, A and KA) compared to sham-treated controls (group P). At the same time CD3+ expression was lowest in the group exposed to acrolein alone (group A). As a result, the pulmonary Foxp3/CD3 ratio was highest in the group receiving acrolein alone, followed by mice to which KLH and acrolein was co-applied. The Foxp3/CD3 ratio in mice sham-treated with PBS or treated with KLH alone was significantly lower compared to the acrolein-exposed groups.

Thus, acrolein exposure during allergic sensitization significantly altered the capability to mount an adequate humoral and cellular immune response. Furthermore, the increase in the Foxp3/CD3 ratio pointed towards a trade-off towards a more tolerogenic environment. Though acrolein had been only applied via the nasal mucosa, it affected local and systemic immunity.

### Prior exposure to acrolein promotes tumor growth

**Figure 4:**
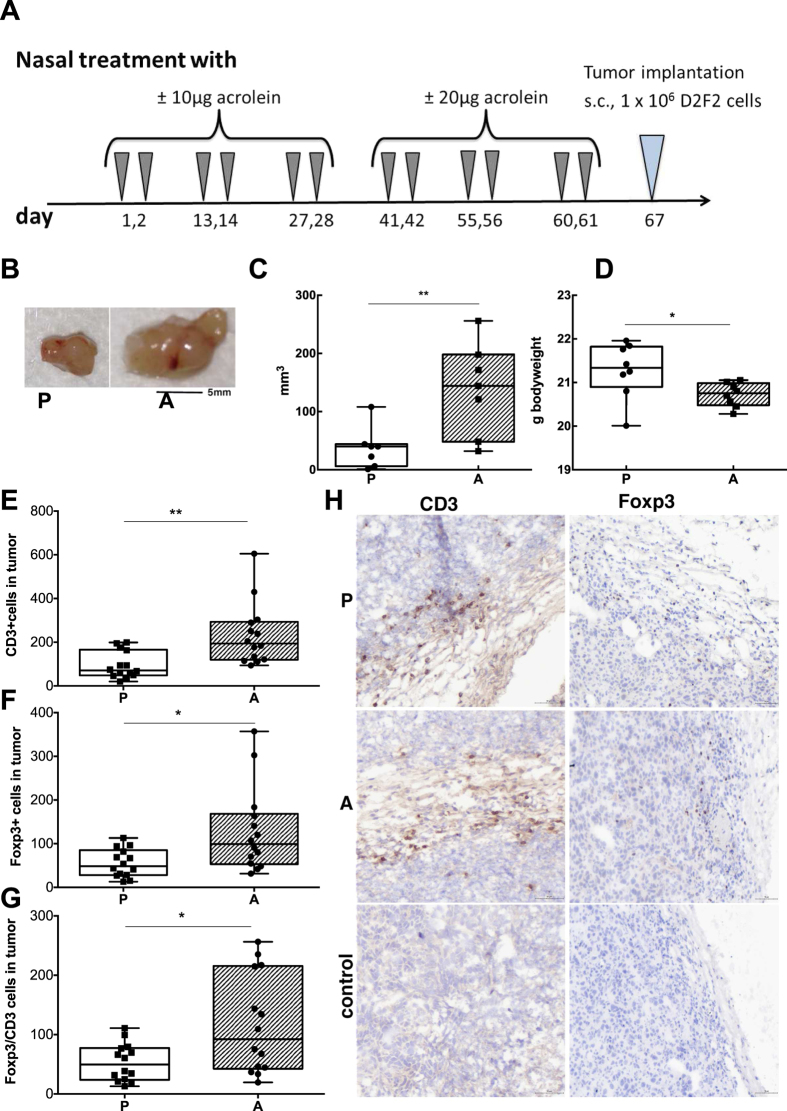
Tumor promotion upon prior exposure to acrolein. (**A**) As depicted in the treatment protocol, mice were intranasally pretreated 6 times in biweekly intervals with acrolein or PBS. After one week interval, murine mammary carcinoma cell-line D2F2 derived from BALB/c mice were injected subcutaneously in the right flank and their growth was monitored. (**B**) Representative pictures of excised tumors of mice previously pre-treated intranasally with PBS (P, n = 8), or acrolein (A, n = 8). Summary of (**C**) tumor size and (**D**) body weight of mice 11 days post-tumor engraftment in the different groups. Whole tumor sections were imaged by TissueFAX and positive stainings were quantified by Histoquest. Summary of immune-histochemical quantification of (**E**) CD3+ cells, (**F**) Foxp3+ cells and (**G**) Foxp3/CD3 ratio of tumor sections in the different treated groups. (**H**) Representative regions of tumor sections stained for CD3, Foxp3 and their respective isotype controls are shown. 2 sections/animal and staining were analyzed by Tissue FAX. Groups were compared by unpaired T test. Mean ± SEM; *P < 0.05; **P < 0.01.

The immune system is pivotal in tumor surveillance and defense^26^, but it is significantly impaired by a strong tolerogenic microenvironment in and around the tumor^27^. To study whether acrolein could shape a tolerogenic environment and thereby pave the way for subsequent tumor growth, mice were treated nasally with acrolein or PBS in biweekly intervals six times, before D2F2 mammary tumor cells were implanted subcutaneously one week later (Fig. 4). By grafting the tumors one week after the last acrolein-treatment, we excluded any direct cancerogenic effect of acrolein. Indeed, acrolein promoted tumor growth in comparison to sham-treated controls. Moreover, tumor growth was consumptive and correlated with a significantly lower body weight in the acrolein-group after 11 days compared to controls (Fig. 4B–D).

Like in the lungs in the allergy model, a significant accumulation of CD3+ and Foxp3+ cells and a higher Foxp3/CD3 ratio was detected in tumors of mice treated with acrolein compared to sham-treated controls (Fig. 4E–H). Hence, acrolein had a persistent pro-tumorigenic effect in our murine model associated with an increased accumulation of intratumoral Foxp3+ and CD3+ cells.

### Acrolein inhibits NF-κB, but activates the AhR pathway

**Figure 5:**
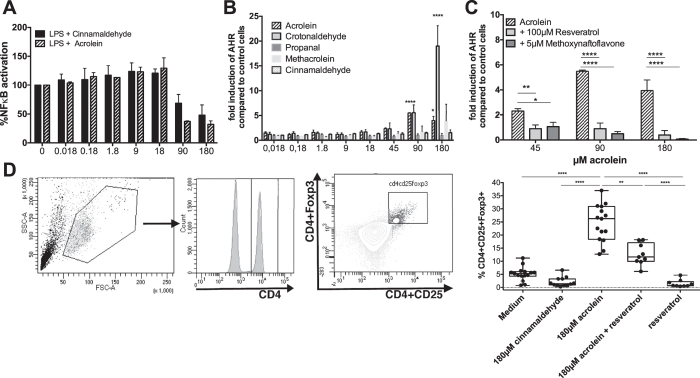
Acrolein promotes Foxp3+ expression via activation of the aryl-hydrocarbon receptor. (**A**) NF-κB inhibition was measured with monocytic THP1-XBlue cells, which were treated with LPS and increasing concentrations of acrolein or cinnamaldehyde for 18 hours. Bars represent summary from two independent experiments normalized to cells stimulated with LPS alone. (**B**) AZ-AhR cells were treated with different concentrations of acrolein or cinnamaldehyde crotonaldehyde, propanal and methacrolein in increasing concentrations (0.018/0.18/1.8/9/18/45/90/180 μM), with (**C**) acrolein alone or in combination with the antagonists resveratrol or 3′-methoxy-4′-nitroflavone for 18 h, before luciferase-activity was measured in the supernatant. Bars represent data from three independent experiments normalized to medium alone for B and two independent experiments for C. Data presented as mean ± SD, Statistical analyses was made using 2way RM ANOVA following Sidak’s multiple comparisons test, respectively. Significances to controls are depicted. (**D**) PBMCs were stimulated for 72 h with cinnamaldehyde, acrolein and or resveratrol. Gating Strategy and summary of % of CD4+CD25+Foxp3+ cells as determined by FACS of 5 independent experiments (n = 15). *p < 0.05, **p < 0.01, ***p < 0.001, ****p < 0.001.

As NF-κB signaling is also pivotal in controlling the proliferation of naïve T cells^28^, inhibition of the NF-κB pathway might contribute to the observed suppressed cytokine release from splenocytes. To address this molecular pathway, we used a monocytic reporter cell line in which acrolein inhibited NF-κB activation in a concentration-dependent manner^29^ (Fig. 5A). Regulatory T cells also express AhR and using a human reporter cell line for AhR^30^ we could demonstrate that acrolein, weaker than the indirubin control (115 ± 1.3 fold induction compared to medium), but concentration-dependently activated AhR. This effect was specific to acrolein. It was not achieved by using its chemical derivative cinnamaldehyde (CA), which was used as control as it is also able to inhibit NF-κB signaling (Fig. 5B)^7^. Hence, we expanded our controls to crotonaldehyde, propanal and methacrolein. Indeed, also crotonaldehyde, but propanal and methacrolein were not able to active AhR in our gene reporter assay (Fig. 5B) indicating that α,β-unsaturated aldehydes are able to activate AhR in the cytoplasm^7^. Moreover, activation of the AhR by acrolein and crotonaldehyde was specific as addition of the AhR-antagonist resveratrol and 3′-methoxy-4′-nitroflavone could inhibit activation (Fig. 5C)^31^,^32^,^33^.

### Foxp3+ induction by acrolein is abrogated by resveratrol

We addressed whether a causative relation between acrolein exposure, AhR activation and regulatory immune cells, could be established in human samples. Therefore, we stimulated human peripheral blood mononuclear cells with increasing concentrations of acrolein alone or in combination with resveratrol as AhR-antagonist for 72 hours and subsequently analyzed their CD4+CD25+Foxp3+ expression. Control cells were stimulated with resveratrol alone, cinnamaldehyde as negative control or indirubin as a strong AhR-agonist. Cells were stained and gated for CD4+, before plotting for CD25+ and Foxp3+. We observed a significant concentration-dependent increase of CD4+CD25+Foxp3+ cells with acrolein (Fig. 5D), that was not seen with cinnamaldehyde, resveratrol or indirubin alone. The observed increase was to a great extent inhibited by the addition of resveratrol (Fig. 5D). CD4+CD25+Foxp3 cells generated by acrolein were atypical, as they did not secrete cytokines (data not shown) and thus appeared to be in an anergic state.

Likely, the integrative sum of non- as well immunological changes like the activation of the AhR pathway besides inhibition of the NF-κB signaling contributes to the observed increase in Foxp3+ expression.

## Discussion

The question why smoking is protective against developing allergies, but positively associated with cancer, has not been answered so far. Instead of using smoke extracts like in other studies, we concentrated in this study on acrolein as a major toxic smoke compound. Our data suggest that acrolein severely suppresses immune responses via activation of Foxp3+ T-regulatory cells with divergent results on the two disease conditions. Our data also suggest that acrolein may exert immunosuppression by activating AhR and blocking this pathway by resveratrol indeed reversed Foxp3+ expression.

Longitudinal studies propose that cigarette smoking prevents allergic sensitization^34^. In cross-sectional studies maternal smoking decreased the risk of allergic sensitization^17^,^35^,^36^,^37^,^38^. We hypothesized that acrolein as a main compound occurring in cigarette smoke could produce systemic immune suppression, thereby affecting specific B- and T-cell responses and inhibit allergic sensitization. In our allergy mouse model indeed acrolein exposure significantly prevented allergic sensitization and immediate type hypersensitivity responses. This was largely due to the fact that mice sensitized against an antigen in combination with acrolein failed in generating antigen-specific antibodies and their immune cells secreted less cytokines^39^.

We considered that T-regulatory cells could be causative for the observed immune deviation, as in our allergy model acrolein led to an accumulation of atypical, anergic Foxp3+ cells in the murine spleen and the lungs. Thus, we attribute the observations of other studies using cigarette smoke showing an increase of regulatory T cells in the murine lung^40^ as well as the suppression of specific T cell responses in humans by cigarette smoke to acrolein^41^. Supporting data also come from cross-sectional studies associating higher regulatory T cell-levels in blood in smokers^42^ and in women exposed to biomass smoke^43^, which are sources in which high doses of acrolein is generated.

There are, however, only few reports that side-by-side compare Treg populations among smokers with intact pulmonary capacity, and non-smokers. A prominent upregulation of CD4+CD25+ T regulatory cells was found in bronchoalveolar lavage fluid and blood of smokers with preserved lung function^44^,^45^, compared with persons who never smoked^46^. Data concerning the numbers of regulatory T cells in patients with chronic obstructive pulmonary disease, COPD, are more diverse, depending on the stage of disease^44^,^46^,^47^,^48^. The numbers of Foxp3+ cells may also vary in large or small airways of smokers and COPD patients and correlate with smoked packs/year^49^. Overall, the published data suggest that while smokers with preserved lung function have increased regulatory T cell -levels, a more complex picture emerges, when the lung function is affected.

Smoking is associated with moderate to heavy exposure to acrolein over time and clearly increases the likelihood to develop cancer. While the probability to die from lung cancer before age 75 is for non-smoking females and males 0.2 or 0.4%, this risk increases dose-dependently to 5.5 or 2.6% for former smokers, 15.9 and 9.5% for current smokers and 24.4 and 18.5% for heavy smokers^50^. Based on the results from our allergy mouse model we anticipated that the acrolein-associated immune suppression could be detrimental in cancer, as it is generally accepted that in many cancer types like breast cancer^51^, hepatocellular^52^ and gastric cancer^53^ an immunosuppressive tumor microenvironment and the extent of intratumoral Foxp3+ expression correlates with disease progression and poor prognosis^54^,^55^,^56^. In our BALB/c mouse tumor model using syngenic D2F2 mammary cancer cells as a model of any type of cancer, acrolein exposure resulted in enhanced tumor growth associated with an accumulation of intratumoral Foxp3+ cells. We thus propose that acrolein acts tumor promoting in an antigen-independent manner by fostering the immunosuppressive cancer microenvironment. Interestingly, the intranasal acrolein application in the mice resulted in a systemic immunosuppression with sustained effect even after acrolein “cessation”, corresponding to the setting of “former smokers” and their higher cancer risk. However, we are aware that we concentrated on acrolein *per se*, as a single cigarette smoke compound rather than on tobacco smoke with all different constituents like for instance in previous models of COPD^57^,^58^.

Due to the chemical similarity we decided to use cinnamaldehyde as control substance, but only acrolein was able to promote Foxp3+ expression on human immune cells. Acrolein is known to block NF-κB signaling similar like its chemical derivative cinnamaldehyde^29^ via its reactive unsaturated aldehyde by preventing oligomerization of TLR4 on the plasma membrane^59^. Consequently, we went in search for a possible cytosolic interaction partner for acrolein and found AhR that –with other ligands - has already been associated with suppression of Th2 cell differentiation^60^,^61^ and increased numbers of CD4+CD25+Foxp3+ cells^61^ *in vivo*. Using an AhR- reporter cell line^30^, we were able to show that acrolein concentration-dependently activated AhR and that activation was antagonised by resveratrol, as well as by the Ahr antagonist 3′-methoxy-4′-nitroflavone. Further studies with similar compounds like crotonaldehyde, propanal and methacrolein revealed that indeed a free α,β-unsaturated structure of the aldehyde is essential to activate AhR. Propanal which lacks the double bond and the methyl group on the *a* carbon atom of methacrolein hindered AhR-activation. Cinnamaldehyde was not able to activate AhR, despite its free α,β-unsaturated structure as it did not readily cross the plasma membrane and hence was not able to activate AhR. AhR-expression levels vary within immune cells. Regulatory T cells, besides other immune cells, express the AhR^62^ which therefore may contribute to immune homeostasis. In this regard, the differences seen in various studies upon addition of acrolein acting either as a suppressor^23^ or as exacerbator^22^ could be explained by the applied doses and immune status of the study subjects. In both disease models of our study, allergy and cancer, and using moderate acrolein amounts, acrolein purely turned on immune suppressive mechanisms.

The postulated acrolein-AhR-immune regulation axis could be further affirmed by our *in vitro* studies using human blood mononuclear cells, when Foxp3+ expression could be antagonized by resveratrol. Resveratrol is a natural phenol occurring in many fruits and plants^63^, which gained special attention as an anti-cancer agent, in several clinical trials^63^.

The present study has some limitations that deserve comment. First, in our study we concentrated on acrolein. However, we are aware that also other smoke compounds are able to contribute in immunosuppression or may counteract the impact of acrolein. Second, we simplified the sensitization route to the nasal mucosa to avoid aerosolized acrolein which would also be encountered via the skin. This resulted in a relatively high concentration of acrolein with irritative potential at the nasal mucosa of mice during the applications, even though applied dose of acrolein corresponded to levels relevant in passive smoking. The immune effects in mice were, however, fundamental and systemic, and data observed in human smokers and non-smokers validate our model^17^,^34^,^35^,^36^,^37^,^38^,^44^,^45^,^46^. Last, the molecular mechanisms were eluded by “*in vitro*” studies with reporter cells and antagonists and, therefore, future studies must clearly confirm the specific effects of acrolein on the promiscuous AhR receptor in the light of other receptor candidates. We believe that, by knowing its path of action, therapeutic and prophylactic strategies may emerge to counterbalance the impact of acrolein.

In conclusion, our data add to the current understanding of how acrolein from smoke manipulates our immune system, preventing allergic sensitization on the one hand and promoting tumor growth on the other hand.

## Materials and Methods

### Animals

Female BALB/c mice, 8–10 weeks of age, were obtained from Charles River Laboratories and kept under conventional housing and treated according to European Union rules of animal care with the permission of the Austrian Ministry of Sciences (BMWF 66.009/0283-II/3b/2011 and 66.009/0313-II/3b/2011).

### Cells

AZ-AhR cell line are human hepatoma HepG2 cells, which were transfected with pGL-4.27-DRE construct containing several AhR binding sites upstream of luciferase reporter gene^30^. Reporter plasmid pGL-4.27-DRE was constructed as follows: Two copies of F site sequences and one copy of B site and D site sequences of mice Cyp1a1gene13 were synthesized and inserted using KpnI-XhoI enzymes into the multiple cloning region of pGL4.27 [luc2P/minP/Hygro] vector (Cat.# E8451) from Promega (Hercules, CA), upstream of the minimal promoter and luc2P reporter gene sequence. Responsive clones were obtained by selection on Hygromycin. AZ-AHR were cultured in Dulbecco’s modified Eagle’s medium (DMEM) supplemented with 10% of fetal calf serum, 100 U/ml streptomycin, 100 μg/mL penicillin, 4 mM L-glutamine, 1% nonessential amino acids, and 1 mM sodiumpyruvate. Cells were maintained at 37 °C and 5% CO2 in a humidified incubator. The mutational status was not tested. THP1-XBlue are commercially available from Invivogen. D2F2 mammary carcinoma cells a kind gift from Prof. Wei-Zen Wei (Karmanos Cancer Institute, Wayne State University School of Medicine, Detroit, Michigan, USA). Cells are routinely tested for mycoplasma contaminations.

### Allergic sensitization and challenge of mice

Each group consisted of eight animals (age: 8–10 weeks), except of the sham-treated group, which received PBS and consisted of 4 animals (group P). Results of two separate, independent experiments were compared.

Mice were nasally immunized 5 times in biweekly intervals. For the first three cycles 5 μg monomeric Keyhole limpet hemocyanin (KLH, Biosyn^®^) (group K) and/or 10 μg acrolein (group A) in 10 μL of phosphate buffered saline (PBS) on 2 consecutive days were administered, whereas for cycle 4 and 5 10 μg KLH and/or 20 μg acrolein (Sigma 01679) in 20 μL of PBS on 2 consecutive days were administered.

One week later mice were nasally challenged with 10 μg KLH on two consecutive days, before conducting whole body plethysmography (WBP) to determine lung function. The next day mice were challenged intraperitoneally with 50 μg KLH in PBS. The animals were monitored for anaphylactic symptoms and rectal temperatures were recorded after 20 minutes.

Animals were subsequently euthanized by gradually introduction of CO_2_. Thereafter, blood was collected by cardiac puncture. Serum was stored at −80 °C until further processing. Spleens and lungs were collected. Lungs were perfused with PBS, before they were fixed in 3.7% neutral paraformaldehyde overnight and embedded in paraffin. Paraffin-sections were cut to 5 μm thickness using a microtome (Histocom).

### Tumor grafting in mice

Acrolein or PBS were nasally applied on two consecutive days 6 times (round 1–3: +/−10 μg acrolein, round 4–6: 20 μg +/− acrolein/mouse) in biweekly intervals as described in the sensitization section (n = 8/group). One week later, murine mammary carcinoma cell-line D2F2 derived from BALB/c mice (1 Mio cells/mouse) were injected in the right flank subcutaneously. The optimal cell density for engraftment of D2F2 cells, allowing solid nesting of the tumor cells on the one hand, but not resulting in too rapid growth on the other, was determined in pre-experiments (data not shown). D2F2 cells were kindly provided by Prof. Wei-Zen Wei (Karmanos 135 Cancer Institute, Wayne State University School of Medicine, Detroit, Michigan, USA)^64^. Tumor development and body weight of mice were controlled daily by serial measurements of tumor size; the tumor volume was calculated according to the following equation: tumor volume (mm^3^) = *d*^2^ × *D*/2, with *d* as the shortest and *D* as the longest diameter as previously described. Animals were euthanized when the tumor reached a volume of more than 300 mm^3^. Tumor sections were embedded in OCT compound and 5 μm thick frozen sections were cut.

### Measurement of antigen-specific immunoglobulins

KLH-specific IgG1, IgG2a, IgE, and IgA were measured by ELISA. Briefly, KLH (0.1 μg/well) or serial dilutions of mouse IgG1, IgG2a, IgE, and IgA standards (highest concentration for all standards: 100 ng/ml) were coated, blocked with 1% BSA in PBS, and incubated with diluted sera (1: 100 for IgG1, IgG2a, IgG2b and 1:10 for IgE) overnight. Detection was performed by monoclonal rat anti-mouse IgG1 (cloneA84–1), IgG2a (clone R19-15) IgG2b (clone R12-3), IgA (clone c10-1), or IgE (clone R35-72) followed by polyclonal peroxidase-labeled goat anti-rat IgG IgG (GE Healthcare). All primary antibodies were from BD Pharmingen. Tetramethylbenzidine (BD Biosciences) was used as substrate. The reaction was stopped with 1.8 M sulfuric acid and detected at 450 nm.

### *In vitro* cytokine responses

Splenocytes were plated at a density of 5 × 10^6^ cells/ml in 48-well cell culture plates (ThermoScientific) and cultured with 5 and 25 μg/ml KLH, 0.5 μg/ml acrolein, 2.5 μg/ml concanavalin A (Sigma), or medium alone for 72 h at 37 °C/5% CO_2_. Subsequently, cytokines of cultured supernatants were measured by ELISA (eBiosciences, for IL10, IL13, IFNg and IL5; R&Dsystems for BAFF) according to the manufacturer’s instructions.

Similarly, supernatant of stimulated human PBMCs were assessed for IL10 (ebioscience) and IL6 (ebioscience). ELISAs for human IL13 and IFNγ have a reported sensitivity of 4 pg/ml, for human IL10 and IL6 the reported sensitivity is 2 pg/ml.

### Flow cytometric analysis of CD4+CD25+Foxp3+ cells in spleens

For the evaluation of Treg cells, single-cell suspensions of splenocytes were stained using the Anti-Mouse/Rat Foxp3 Staining Set PE of eBioscience. Briefly, cells were fixed, permeabilized and stained according to the manufacturer’s protocol (eBiosciences) using anti-forkhead-box-protein 3 (FOXP3) PE (eBioscience, clone FJK-16s), anti-CD4 FITC (clone RM4-5) and anti-CD25 APC (clone PC61.5) antibodies. Cells were acquired on a flow cytometer and gated on CD4+ in the living population, before gating on CD25+Foxp3+ cells. Treg numbers per spleen were calculated by multiplying % of CD4+CD25+Foxp3+ cells with the total numbers of isolated cells per spleen.

### Immunohistochemistry

#### Lung and tumor sections were stained for CD3 and Foxp3

Lung sections were stained for CD3 as previously described. Briefly, after deparaffinization, antigen retrieval (30U/ml Proteinase K from Sigma #4850 for 15 min at 37 °C), blocking of endogenous peroxidase (3% H_2_O_2_ in Methanol, 10 min), permeabilization with 0.2% Tween in PBS and further blocking of unspecific binding with blocking serum (ABC kit, Vectastain), anti-CD3-antibody (1:50 in 1.5% goat serum, AbD Serotec, USA, MCA 1477) or isotype control, rat IgG2b was added overnight at 4 °C, before further incubation with biotinylated anti-rat IgG in blocking serum (30 min) and avidin-HRP (30 min). After washing Dako Chromogen System was used as a substrate and reaction was stopped with water. Between each step vigorous washing was performed with PBS.

For Foxp3, slides were deparaffinized and heat-mediated antigen retrieval with 10 mM sodium citrate, pH 6.0 for 20 minutes was performed. After cooling down slides were blocked for endogenous peroxidase (3% H_2_O_2_ in Methanol, 10 min), permeabilization with 0.2% Tween in PBS and for unspecific binding with 1.5% blocking serum (rabbit ABC staining system, Santa Cruz) and incubated with rabbit anti-mouse Foxp3 (1:400 in 1.5% goat serum)(1.98 mg/ml) or isotype control, rabbit IgG, overnight at 4 °C. Subsequently biotinylated goat anti-rabbit IgG was applied for 30 min and thereafter Avidin-horseradish peroxidase (Avidin-HRP, Vector Laboratories, 1:250) was added to the slides (30 min). Dako chromogen system was used as a substrate. Reaction was stopped with tap water.

For determination of CD3 in frozen sections of tumors, slides were fixed with aceton, blocked for endogenous peroxidase with 0.3% H_2_O_2_ in PBS and for unspecific binding with 1.5% goat serum before adding antiCD3 (Serotec, MCA 1477) or isotype control (ratIgG1, MCA6004GA), followed by incubation with biotinylated anti-rat IgG and Avidin-HRP (ImmunoCruz™ rat ABC Staining System sc-2019). As substrate Dako chromogen system was used. Between each step vigorous washing with PBS was performed.

Similarly, for Foxp3 staining in frozen sections, after fixing with ice-cold aceton, blocking endogenous peroxidase and unspecific binding, slides were incubated with Fix/perm solution (eBiosciences) for 15 min, washed in perm buffer (eBiosciences, 2 × 5 min), anti-Foxp3 (1:8000 in permbuffer, Abcam, ab54501) or isotype control, rabbit IgG (Abcam, ab171870) was added overnight at 4 °C, before further incubation with biotinylated goat anti-rabbit IgG in permbuffer (30 min, ImmunoCruz™ rabbit ABC Staining System sc-2018) and avidin-HRP (30 min, ImmunoCruz™ rabbit ABC Staining System sc-2018). After washing with PBS, Dako chromogen System was applied to slides as substrate and reaction was stopped with water.

All slides were counterstained with haematoxylin (Sigma MHS16) and mounted with fluoromount-G (Sigma F4680). Stainings of slides were acquired using TissueFaxs with Zeiss Axio Imager Z1 microscope magnification x20 and quantified with HistoQuest^®^ cell analysis software from TissueGnostics. To estimate amount of accumulated cells per tumor % of CD3+ and Foxp3+ cells were multiplied with respective tumor volume.

### Aryl-hydrocarbon receptor activity assay

Aryl-hydrocarbon receptor activity was measured using a stably transfected gene reporter human cell line AZ-AhR^30^. Briefly, 2 × 10^4^ cells/well were seeded in 96-well plates and incubated for 18 hours at 37 °C and 5% CO_2,_ before cells were stimulated 18 h in triplets with acrolein, cinnamaldehyde, crotonaldehyde, propanal and methacrolein (all from Sigma) in increasing concentrations (0.018/0.18/1.8/9/18/45/90/180 μM) was used as negative control, whereas indirubin (0.03 μg/ml, Sigma) was used as a positive control and as AhR-antagonists resveratrol 100 μM (Sigma) and 3′-methoxy-4′-nitroflavone 5 μM (Sigma). After adding lysis buffer and a single freeze-thaw cycle, 20 μl/well of lysates were transferred into a black 96-well flat-bottom plate (Thermo Scientific) and bioluminescent reaction were started with addition of 100 μl/well of luciferase assay reagent (Promega). Chemiluminscence was measured (10 sec/well) using a spectrophotometer Tecan InfiniteM200 PRO.

### NF-κB/AP-1 activation assay

NF-κB activation assays were performed using THP1-XBlue reporter cells, stably expressing an NF-κB/AP-1-inducible secreted alkaline phosphatase reporter (SEAP), as described previously^29^. THP1-XBlue cells were were stimulated with lipopolysachharide (LPS from Sigma) and increasing concentrations of acrolein or cinnamaldehyde. In brief, 1 × 10^5^ cells/well were seeded into a 96-well plate and stimulated with acrolein or cinnamaldehyde (0 to 180 μM) alone or in combination with LPS E coli 055:B55 (5 μg/ml, 15 000 EU/ml) for 18 h. NF-κB activity was determined adding QUANTI-Blue (Invivogen rep-qb1), as a substrate of secreted alkaline phosphatase in the supernatants and further incubation for 8 h at 37 °C and 5% CO_2_. Subsequently, optical density (OD) was measured at 625 nm using a Tecan InfiniteM200 PRO spectrophotometer.

### Stimulation of PBMCs

Human blood donation of volunteers were approved by the institutional ethics committee of the Medical University of Vienna and conducted in accordance with the Helsinki Declaration of 1971. All subjects gave their full written informed consent. Fifteen volunteers donated 15 ml blood for isolation of peripheral blood mononuclear cells by Ficoll-Paque (GE Healthcare) as already described. Isolated PBMCs (0.5 Mio/ml) were incubated with increasing concentrations of acrolein (36, 108, 180 μM) acrolein, 180 μM cinnamaldehyde, 80EU/ml LPS E coli 055:B55, 200 nM indirubin and/or 100 μM resveratrol.

For the evaluation of Treg cells, single-cell suspensions of splenocytes were stained using the Anti-Human Foxp3 Staining Set of eBioscience #88-8998-40. Briefly, cells were fixed, permeabilized and stained according to the manufacturer’s protocol using an anti-CD4FITC (clone RPA-T4), anti-CD25 PE (clone BC96) cocktail and subsequent staining with anti-forkhead-box-protein 3 (FOXP3, clone PCH101) APC antibodies. Cells were acquired on a flow cytometer and gated on CD4+ in the living population, before gating on CD25 + Foxp3+ cells.

### Statistics

Statistical analyses when comparing more than two groups were calculated by ANOVA following Newman-Keuls Multiple Comparison test using GraphPad Prism 5 software. Differences between two groups were analyzed using unpaired *t*-test. Differences in the concentration-dependency of NFκB and AhR-activation upon acrolein or cinnamaldehy stimulations were statistically analyzed using 2way RM ANOVA following Sidak’s Multiple Comparison test. The data were expressed as mean ± SEM. A value of *P* < 0.05 was considered significant.

## Additional Information

**How to cite this article:** Roth-Walter, F. *et al*. Janus-faced Acrolein prevents allergy but accelerates tumor growth by promoting immunoregulatory Foxp3+ cells: Mouse model for passive respiratory exposure. *Sci. Rep.* **7**, 45067; doi: 10.1038/srep45067 (2017).

**Publisher's note:** Springer Nature remains neutral with regard to jurisdictional claims in published maps and institutional affiliations.
